# The necessity of improving cardiovascular health in commercial motor vehicle drivers

**DOI:** 10.1016/j.ahjo.2022.100206

**Published:** 2022-09-16

**Authors:** Judy Kim, Chloe R. Duvall, Roger S. Blumenthal, Nadia R. Sutton

**Affiliations:** a Division of Cardiovascular Medicine, Department of Medicine, Michigan Medicine, Ann Arbor, MI, USA; b Department of Medicine, Johns Hopkins University School of Medicine, Baltimore, MD, USA; c Division of Cardiology, Johns Hopkins Ciccarone Center for the Prevention of Cardiovascular Disease, Baltimore, MD, USA

**Keywords:** Cardiovascular disease, Prevention, Occupational health, Diabetes, Hypertension

## Abstract

Commercial motor vehicle (CMV) drivers have an increased risk for cardiometabolic risk factors and higher rates of cardiovascular disease relative to the general population. Lifestyle factors, including the sedentary nature of driving and lack of access to healthy foods, contribute to the disproportionately high cardiovascular disease (CVD) risk among commercial vehicle (CMV) drivers. Occupational physicals represent an important opportunity to reach populations with a high prevalence and incidence of coronary artery disease who may not otherwise seek preventive care. These visits are an opportunity to discuss primary and secondary prevention, including lifestyle topics such as diet and exercise, and potentially recommend or implement preventive medical therapy when indicated. Future iterations of licensing guidelines for drivers should seek to incorporate updated recommendations regarding diet, physical activity, and preventive pharmacologic therapies.

Commercial motor vehicle (CMV) drivers have an increased risk for cardiometabolic risk factors relative to the general population (Fig. 1) [1], [2], [3], [4], [5], [6], [7], [8]. In the United States, CMV drivers include heavy truck and passenger bus drivers, who have a commercial license issued by the Federal Motor Carrier Safety Administration (FMCSA). Taxi and rideshare drivers do not require licensing by the FMCSA. Drivers, including bus and truck drivers, often spend many hours of their work day sedentary, with one study finding increasing rates of obesity among drivers with higher numbers of miles per year and more hours spent behind the wheel per day [9]. Commonly, the most accessible dietary options are high in sugar and saturated fat. The work hours associated with shift work are associated with fatigue and physical inactivity [10]. Exposure to harmful chemicals from exhaust [11] and tobacco use [2] are other occupational hazards faced by CMV drivers.

**Fig. 1:**
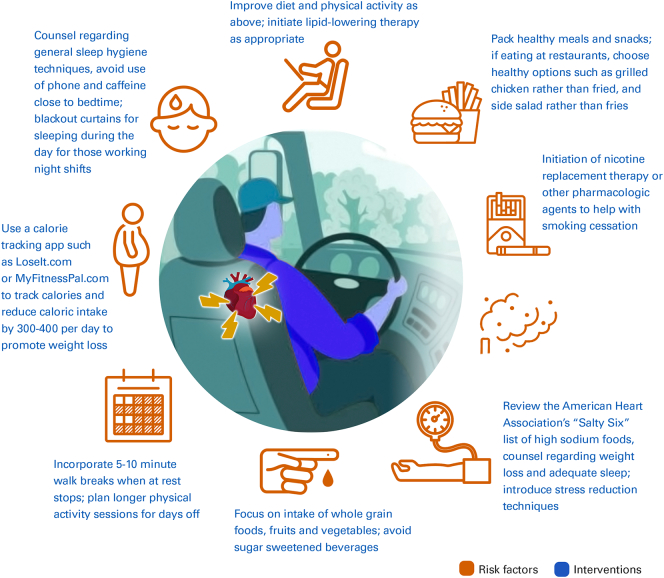
Lifestyle factors impacting cardiovascular disease risk factors among commercial vehicle drivers Lifestyle factors, including the sedentary nature of driving and lack of access to healthy foods, contribute to the disproportionately high cardiovascular disease (CVD) risk among commercial vehicle (CMV) drivers. Workplace exposure to harmful fumes and long work hours leading to dysregulated sleep schedules likely also contribute to high rates of dyslipidemia, hypertension, obesity, insulin resistance, and, ultimately, CVD risk observed among CMV drivers.

Historically, studies have focused on health behaviors of long-haul truck and other CMV drivers; there is a paucity of data on major adverse cardiovascular (CVD) outcomes [12] attributable to the occupation itself. Bigert et al. found that bus drivers were 2.1 times more likely than controls to experience a myocardial infarction [12]. This was no longer significant after adjustment for certain lifestyle factors and comorbidities, suggesting that these factors play a large role in the excess risk observed in this group. Census data demonstrate that CMV drivers have lower educational attainment and lower rates of health insurance compared to the general population [13], suggesting that lower socioeconomic status contributes to cardiovascular risk in this group. Research strongly supports a relationship between health behaviors, socioeconomic status, and CVD outcomes in the general population [14], [15], as well as in CMV drivers [16].

For those providing transportation to large groups, the consequences of a myocardial infarction or stroke that could cause driver incapacitation are dire. Loss of control of a vehicle could also result in catastrophic consequences for bystanders not within the vehicle. Regulatory agencies are charged with considering the health of the CMV driver as well as risk of their driving on the general population. Detailed guidelines for screening and treatment of CAD therefore exist for these groups [17], [18]. This topic is the subject of a recently published article discussing the current guideline recommendations [19].

Occupational physicals represent an important opportunity to reach populations with a high prevalence and incidence of coronary artery disease who may not otherwise seek preventive care. There are 3.5 million commercial truck drivers in the U.S. [13], which represents an important opportunity to reach a large number of at-risk individuals. These visits are an opportunity to discuss primary and secondary prevention, including lifestyle topics such as diet and exercise, and potentially recommend or implement preventive medical therapy when indicated. Harnessing these existing and required periodic visits as an opportunity for primary or secondary prevention could have a widespread, positive impact on the cardiovascular health of those requiring licensure. Implementation of preventive measures at the time of an occupational evaluation is likely to improve cardiovascular outcomes.

## 1 Licensing requirements

Guidelines require CMV drivers to undergo physical exams every two years in order to be officially licensed [19], with the goal of assessing cardiovascular risk and recommending interventions as appropriate. Risk assessment is important for the general population to aid in clinician-patient discussions about CVD prevention and allocate pharmacologic therapy when indicated [20]. It carries added importance for CMV drivers, given high baseline rates of CVD risk factors [1], [2] and the high risk of mortality should a driver become incapacitated due to a cardiac event while driving.

Current guidelines require drivers to self-report any symptoms that may potentially have an effect on their ability to safely carry out their occupation. Additional screening and, in some cases, interventions are required prior to licensure. Of note, while the Federal Motor Carrier Safety Administration (FMCSA) guidelines require symptomatic drivers undergo evaluation by a cardiologist, the recently published chest pain and revascularization guidelines do not make any specific recommendations for high-risk occupations [21], [22]. This could be a helpful addition to future iterations of the guidelines.

Additionally, given that reporting symptoms could impair a driver's ability to maintain gainful employment, reliance on self-reported symptoms for screening is an inadequate method for identifying symptomatic CAD and predicting future events. The FMCSA could consider adopting a more objective approach to risk stratification as outlined in the 2019 American College of Cardiology/American Heart Association Guideline on the Primary Prevention of Cardiovascular disease [23]. This approach recommends use of the Pooled Cohort Equation [24], assessment of risk enhancing factors, and consideration of coronary artery calcium scoring to estimate risk and guide interventions.

While functional testing, such as exercise treadmill tests, can provide useful information about drivers' ability to carry out the physically demanding aspects of the job, assessment of total atherosclerotic disease burden with anatomic testing, such as coronary artery calcium score, for asymptomatic intermediate risk (≥7.5 % to <20 % 10-year atherosclerotic cardiovascular disease risk) individuals or coronary CT angiography, for symptomatic individuals as an alternative to stress testing, has been shown to more highly correlate with future cardiovascular events than inducible ischemia on functional testing [25], [26]; if functional testing is normal and the patient is not on a statin, a coronary artery calcium scan should be considered if the patient is reluctant to go on a lipid lowering agent.

Current FMCSA guidelines require drivers to be free of ischemia to return to driving, if the presence of coronary artery disease is established. These recommendations are at odds with the findings of the ISCHEMIA trial [27], which suggest that there is no reduction in mortality with an invasive approach for stable ischemic heart disease with an ejection fraction >35 % and no significant left main coronary artery disease. Under the current paradigm, emphasis is placed on reducing the burden of ischemia via coronary artery bypass grafting or percutaneous coronary intervention.

After a prescribed waiting period, drivers may then reapply to return to driving, with input from the referring cardiologist. This also applies to asymptomatic drivers with ischemia. Drivers must then be periodically monitored by an occupational health provider and a cardiologist with yearly exams and an exercise treadmill test every two years. Importantly, if risk factors were instead adequately addressed at the outset and controlled in early to middle-age, there is a much stronger chance that drivers approaching retirement age would be able to maintain their employment, rather than losing licensure due to unrevascularizable ischemia.

## 2 Personalized preventive guidance

Utilizing occupational health medical exams performed for drivers as an opportunity to address cardiovascular risk factors and promote healthy lifestyle tips could provide improved health care efficiency and also provide care to those that would otherwise not seek preventive care independently of the occupational visit. Providers could use the American Heart Association's (AHA) “Life's Essential 8” framework [28], with emphasis placed on counseling CMV drivers to overcome workplace-specific challenges (Table 1 and Fig. 1).

**Table 1:** Challenges and opportunities for promotion of cardiovascular health in commercial drivers [33].

Challenge	Recommendation	Solution
Lack of readily available heart-healthy food options; preponderance of fast-food	Focus on a healthy pattern of eating that includes whole grain foods, fruits and vegetables, lean proteins, nuts and seeds	Pack healthy meals and snacks; if eating at restaurants, choose healthy options such as grilled chicken rather than fried, and side salad rather than fries
Sedentary nature of driving and long work hours lead to fatigue that limits physical activity	Aim for 150 min of moderate or 75 min of vigorous physical activity per week	Incorporate 5–10 min walk breaks when at rest stops; plan longer physical activity sessions for days off
High rates of cigarette/tobacco use; second-hand smoke exposure; boredom, fatigue, and stress while driving trigger urge to smoke	Avoid smoking traditional cigarettes, e-cigarettes, and vaping	Initiation of nicotine replacement therapy or other pharmacologic agents to help with smoking cessation
Long work hours and shift work lead to inadequate sleep	Obtain 7–9 h of sleep per night	Counsel regarding general sleep hygiene techniques, including avoiding use of phone and caffeine close to bedtime; blackout curtains can be helpful for sleeping during the day for those working night shifts
Easy access to calorie dense fast food and lack of physical activity lead to weight gain	Achieve and maintain a healthy body weight and body mass index	See above tips for diet and physical activity; use a calorie tracking app such as LoseIt.com or MyFitnessPal.com to track calories and reduce caloric intake by 300–400 per day to promote weight loss
Intake of foods high in saturated fat and cholesterol raises low density lipoprotein and triglyceride levels	Achieve cholesterol goals	Improve diet and physical activity as above; initiate lipid-lowering therapy as appropriate
Intake of processed, high-glycemic index foods and beverages leads to insulin resistance and diabetes	Achieve blood sugar control	Focus on intake of whole grain foods, fruits and vegetables; avoid sugar sweetened beverages
Intake of high sodium restaurant foods, lack of adequate sleep, and work-related stress exacerbates hypertension	Achieve optimal blood pressure	Review the American Heart Association's “Salty Six” list of high sodium foods, counsel regarding weight loss and adequate sleep as above; introduce stress reduction techniques [34]

The AHA recommends emphasize intake of fruits, vegetables, whole grains, lean protein, and legumes, while minimizing intake of foods with high amounts of added sugar or sodium, red meat, processed meat, and refined grains [29]. Occupational health professionals should counsel CMV drivers that their occupation predisposes them to eating at fast food restaurants and encourage drivers to plan healthy meals along their route or to pack healthy meals. The AHA also recommends a minimum of 150 min of moderate aerobic exercise or 75 min of vigorous exercise per week, with some studies suggesting that this recommendation is inadequate to reduce the development of cardiovascular disease [23], [30]. Health professionals could advise commercial drivers to aim for 10 min of brisk walking each time they stop for fuel and emphasize the importance of prioritizing time for physical activity on days that they are off from work.

Occupational health visits are also an opportune time to screen for diabetes, hypertension, and hyperlipidemia, and for introduction of optimal medical therapy for primary or secondary prevention [31]. While it would be preferable to reduce cardiovascular disease prior to its development, once coronary artery disease develops, secondary prevention measures must be implemented to reduce progression.

Several large studies have found that rates of achieving low-density lipoprotein cholesterol and blood pressure goals and rates of adherence to medications such as statins, antiplatelet agents, and beta blockers range from one-half to two-thirds among enrolled patients, which is unacceptably low for a high-risk cohort [32]. Statistics regarding rates of achievement of optimal medical therapy among those in high-risk occupations are not available, which represents a gap in knowledge and a potential opportunity for improving care efficiency. Streamlined guidelines regarding indications for these medications could also be compiled and shared with occupational health professionals.

## 3 Putting it into practice

Occupational health visits for CMV drivers represent an important opportunity for implementation of both primary and secondary prevention targets aimed at reducing the burden of atherosclerotic cardiovascular disease in a population at high risk for development of cardiovascular disease. For those in high-risk professions such as commercial drivers, the stakes are high from a public safety perspective, and on an individual level, for drivers seeking continued employment. Occupational health professionals could play a more prominent role in promoting a health, so that CMV drivers can remain employed until they chose to retire.

Undoubtedly, the additional time spent in preventive counseling during occupational health visits would require additional human and economic resources. Maintenance of employment and fewer health care expenditures for complications of cardiovascular disease are desirable and on a societal level would likely offset the cost of additional preventive healthcare during occupational visits. Future iterations of licensing guidelines for drivers should seek to incorporate updated recommendations regarding diet, physical activity, and preventive pharmacologic therapies.

## Funding

This research did not receive any specific grant from funding agencies in the public, commercial, or not-for-profit sectors.

## CRediT authorship contribution statement

**Judy Kim:** Writing – original draft. **Chloe R. Duvall:** Writing – original draft. **Roger S. Blumenthal:** Conceptualization, Writing – original draft. **Nadia R. Sutton:** Conceptualization, Writing – original draft, Supervision.

## Declaration of competing interest

The authors declare the following financial interests/personal relationships which may be considered as potential competing interests: Dr. Sutton has served as an advisor to Abbott, Cordis, Shockwave, and Philips, and has received honoraria for speaking from Zoll, Shockwave, Cordis, and Abbott. All other authors have no disclosures.
